# Pervious Concrete Made with Recycled Coarse Aggregate and Reinforced with Date Palm Leaves Fibers

**DOI:** 10.3390/ma16237496

**Published:** 2023-12-04

**Authors:** Adil Tamimi, Sami W. Tabsh, Magdi El-Emam

**Affiliations:** Department of Civil Engineering, College of Engineering, The American University of Sharjah, Sharjah P.O. Box 26666, United Arab Emirates; atamimi@aus.edu (A.T.); stabsh@aus.edu (S.W.T.)

**Keywords:** pervious concrete, permeability, porosity, strength, sustainability, recycled concrete aggregate, fibrous concrete

## Abstract

This study considers 12 pervious concrete mixes incorporating 100% recycled coarse aggregate from old concrete demolition waste and containing various amounts of natural fine aggregate and date palm leaves fibers. First, the properties of the recycled aggregate in terms of their particle size distribution, abrasion resistance, crushing values, specific gravity and water absorption are obtained. Next, the pervious concrete density, compressive strength, tensile strength, permeability and porosity are determined by experimental testing following the relevant standards. The results are analyzed and compared to determine the influence of using recycled coarse aggregate in the mixtures and the impact of the amount of natural sand and volume fraction of the fibers on the mechanical properties, permeability and porosity of the concrete. Findings of the study showed that the use of recycled coarse aggregate in pervious concrete without fine aggregate reduced the compressive strength by 36% and tensile strength by 57%. Replacing 11.7% of the recycled coarse aggregate with natural sand and adding date palm leaves fibers in an amount equivalent to 0.64% volumetric content to such concrete helped increase the compressive strength by 16.2% and tensile strength by 3.2% above the corresponding strengths of the control mix. There is a clear relationship between permeability and porosity due to their correlation with the density of pervious concrete, and the effect of porosity on tensile strength is more influential than it is on the compressive strength. An equation that can predict the tensile strength of pervious concrete from the compressive strength is proposed, as a function of the natural fine aggregate fraction of the coarse aggregate and volumetric content of natural fibers. Results of the research confirm the feasibility of using recycled aggregate in pervious concrete mixes and the positive impact of natural fibers on the mechanical properties.

## 1. Introduction

Pervious concrete (PC), also known as porous or permeable concrete, is a structural type of concrete that possesses a relatively large void content ranging between 15 and 35%, which allows fluids and gases to pass through them, thus eliminating ponding potential on the surface. The high permeability of PC has a somewhat negative effect on the mechanical properties, which can be improved by adding fibers. Ideal usage of PC can be found in road pavements, parking areas and walkways. Such applications reduce stormwater runoff and regenerate the groundwater at a faster rate than usual, which makes the material sustainable and environmentally friendly. 

The use of pervious pavements dates back to the time of the Roman Empire when the builders used cobble and flag stones with voids in between them to enable falling rain to leak through the road to the underground. Later on, the first concept of embedding porosity in paving stones was introduced in the 1800s in European cities, which appreciated the need for a stormwater runoff solution in highly populated areas that receive a lot of rain annually. After the Second World War, permeable paving started appearing on roadways in North America after such type of construction was looked at favorably by environmental agencies. Nowadays, this pavement system has become widely accepted around the world for solving water runoff problems in place of traditional drainage due to its practicality, economy and eco-friendly nature [1].

In addition to providing a solution to the stormwater runoff problem and recharging aquifers at high rates, PC offers a variety of benefits such as the elimination of drainage systems, safer vehicle drivability during rain, assisting plant roots to grow into trees, and filtering dirty storm water. The drawbacks include weaker concrete, the need for longer curing time, a requirement for specialized contractors, a lack of comprehensive design standards, and the necessity for periodic cleaning of clogged pores.

In general, PC possess almost no slump and can be produced with moderate size coarse aggregate ranging 9.5–12.5 mm and a very limited amount of fine aggregate, with water-to-cement ratios commonly varying between 0.30 and 0.40. The resulting mass density of PC is much smaller than that of conventional concrete, normally ranging between 1400 and 2000 kg/m^3^. Geopolymers, supplementary cementitious materials and admixtures can often be utilized in the production of PC. The resulting concrete typically has 3–30 MPa compressive strength and 0.5–4 MPa tensile strength, containing interconnected pores with a size of 2–8 mm which often corresponds to a 80–750 L/minute/m^2^ drainage rate [2]. 

Although the utilization of coarse recycled concrete aggregate (RCA) from demolition waste in PC mixes enhances the sustainability traits of such concrete by reducing the amount of waste dumped in landfills and protecting natural resources, it can have an adverse effect on its strength and impact on its density and permeability. In particular, the source and condition of the recycled aggregate play an important role with regard to whether the influence of the aforementioned aggregate on the fresh and hardened properties of concrete are mild or severe. 

To help improve the mechanical properties of PC utilizing RCA, fibers can be added to the concrete mixture. Including fibers in concrete decreases the cracking openings and propagation, increases the tensile strength, and enhances the overall energy absorption and integrity. Although in recent years there have been extensive studies on concrete utilizing steel, glass, or other synthetic fibers, applicable research on natural fibers still lags behind. The use of natural fibers, such as wood, coconut, bamboo or date palm leaves, in construction materials has a great advantage because fibers are locally present in abundance, can be economically processed, do not require high energy to produce, and have minimal negative impacts on the environment.

A comprehensive literature review on the subject, presented in the next section, showed limited research work has been conducted concerning PC made with recycled materials and including natural fibers. This scarcity of publications on the subject could be because of the high variability associated with recycled concrete due to the influence of the quality of the material being processed by the recycling plant. Therefore, this study is concerned with the investigation of the compressive and tensile strength properties, as well as the porosity and permeability characteristics, of PC made with locally produced coarse RCA in the UAE, with and without natural fibers from date palm trees, and comparing the response to that of corresponding PC made with coarse natural aggregate (NA). The implementation of date palm fibers (DPF) in the recycled concrete mixes greatly adds to the sustainability element of the study and helps in promoting the UAE’s 2050 vision initiative which aims at making the country the first nation within the region to achieve net-zero emissions by the year 2050.

## 2. Literature Review

There is an abundance of published literature on the characteristics, mechanical properties and durability aspects of PC since this type of concrete has been developed some time ago. For example, there is available literature that addresses plain PC [3,4,5,6,7,8,9,10,11,12,13,14,15,16,17] as well as PC that incorporates supplementary cementitious materials [18,19,20,21,22]. However, research on PC that contains RCA as partial or full replacement of NA remains relatively scarce, especially in combination with fibers that are capable of compensating for the inferior properties of the recycled aggregate. Samples of conducted investigations on PC containing RCA with and without fibers are presented below.

Recent research on PC made with RCA includes the work of El-Hassan et al. [23] who showed that an increase in RCA replacement fraction caused a reduction in workability, an increase in void content, and a reduction in compressive and tensile strengths, although slag incorporation could enhance the mechanical performance. Tensile splitting and flexural strength were linearly correlated with the compressive strength, and abrasion resistance of PC was mainly influenced by the RCA replacement. Vieira et al. [24] found that mechanical properties of pervious recycled concrete decrease with a smaller water/binder (w/b) ratio, there was an increase in the permeability and infiltration rate in pervious concretes with RCA, and replacement of NA by RCA increases the surface abrasion of pervious concrete. Zhang et al. [25] used response surface methodology (RSM) to design the mix proportion of recycled aggregate pervious concrete and found that the Box–Behnken Design approach showed that efficient paste thickness and actual coating thickness are notably influenced by the amount of superplasticizer, viscosity-modifying admixture, and set retarder. For target response based on optimizing models of admixtures, suitable aggregate grading and amount combination of admixtures can be attained. Tests on PC with small coarse aggregate gradation by Chaitanya and Ramakrishna [26] demonstrated 12.5% higher compression and flexure strength than the specimens made with relatively larger coarse aggregate gradation. Replacement of normal aggregate with 25% RCA decreased the compressive strength by 60% and increased permeability by 6.5%. The addition of 8–16% silica fumes increased the compressive strength by 56% and flexure strength by 33% but decreased the permeability by 60%. Yang et al. [27] reinforced pervious concrete made with RCA by paste-coating on the surface and established the association between paste-coating thickness with different size RCA and paste with 5% silica fume fluidity, and that of paste fluidity, leading to proper w/c ratio and superplasticizer dose. The obtained concrete presented outstanding workability, adequate permeability (6.5 mm/s), and reasonable compressive strength (28-day $f_{c}^{\prime }$ = 18.5 MPa). Strieder et al. [28] used experiments to establish that an increase in the content of RCA leads to surge in hydraulic performance and a reduction in compressive strength, while rheology modifying admixture does not contribute to mechanical properties. Compared to results from laboratory experiments, field tests showed lower values of compressive strength and modulus of elasticity, and just about the same infiltration rate and modulus of elasticity. Tijani et al. [29] found that utilizing RCA in PC mixes increased the void ratio and hydraulic conductivity but decreased the density and compressive strength, irrespective of the substitution quantity of cement with sorghum husk ash (SHA). Additionally, compressive strength at 5% SHA replacement was greater than the control mix regardless of the fraction of RCA replacement. For a concrete mix incorporating 100% RCA and 25% SHA, the CO2 emission and production costs were, respectively, 38.23% and 51.29% lower than those of the control mixture. Compared to PC with cement, Gowda et al. [30] observed that alkali activated slag-based pervious concrete had the same workability and tensile strength, but 23% less compressive strength. The addition of RCA to the mix reduced the tensile and compressive strengths but helped increase the porosity of the concrete. El-Hassan et al. [31] concluded that RCA reduced the slump, recycled fine glass (RFG) caused minimal reduction in the slump, and GGBS improved the slump flow response. The mechanical properties and abrasion resistance of PC were deteriorated while the permeability increased with the rise in the replacement of NA by RCA and/or NFA by RFG. 

The use of fibers or geogrids to enhance the properties of pervious concrete has been addressed in the available literature by some researchers. For example, Meng et al. [32] were able to achieve porosity above 20%, a permeability coefficient higher than 4.5 mm/s, flexural strength reaching up to 5 MPa, compressive strength attaining up to 30 MPa, and maximum toughness of 90 Jouls when geogrids were placed at both one-third and two-thirds the depth of the member thickness of PC. Zhu et al. [33] found that permeability of the recycled pervious concrete with fiber was highest when the W/C = 0.30, and pervious concrete with thick polypropylene fiber content of 3 kg/m^3^ exhibited highest strength in flexure of 3.42 MPa and in compression of 21.43 MPa. Juradin et al. [34] reached the conclusion that compaction of PC yielded good pore-related and mechanical properties due to the formation of a viscous layer at the contact surface between the aggregate and the cement matrix. The addition of fibers had no influence on the density, a positive impact on the compressive and splitting tensile strengths of the concrete, and unfavorable consequence on permeability. A study by Wu et al. [35] demonstrated that the addition of basalt fiber content of 4 kg/m^3^ to pervious concrete increased the compressive strength by 39%, reduced the flexural strength by 17%, lowered the permeability coefficient by 42%, and reduced the porosity by 35% when compared to control specimens. Results of a study by Ozel et al. [36] showed that the mechanical properties of PC were improved by the inclusion of steel fibers and downgraded with the polypropylene fiber addition, the compressive strength had a strong correlation with the tensile strengths, the infiltration rate increased with polypropylene fiber inclusion, and weak relation between porosity and permeability for the PC mixtures containing fibers.

With respect to PC made with RCA and fibers, Aliabdo et al. [37] determined that use of polypropylene in PC mixes slightly decreased the concrete’s compressive strength, had a positive effect on the tensile strength, and slowed down the degradation resistance. To improve the performance of recycled pervious concrete, silica fume and styrene butadiene latex can be added. Toghroli [38] found that porosity of pervious concrete increased with the utilization of RCA and addition of fibers, and decreased with partial replacement of cement with SF and nano-clay (NC). Moreover, the compressive strength was negatively affected by the addition of RCA and NC, and positively impacted by the incorporation of SF and fibers. Novak et al. [39] developed fiber-reinforced pervious concrete made with recycled aggregate for airfield pavement applications. In comparison with conventional concrete, results of the study showed that although the material possessed a ductile behavior, it had a low modulus of elasticity. A field study utilizing the concrete as subbase course showed a 20% compaction ratio with flatness deviation within the range of 10–25 mm over 2 m length of the course. Mehrabi et al. [40] noticed that adding RCA in pervious concrete increased the water permeability and void content and lowered the mechanical strength and density and suggested incorporating pumice and NC in mixes to densify the pore system. Incorporation of fiber negligibly affected the porosity and permeability but had a significant effect on the compressive and tensile strengths, especially when RCA was present. Xiao et al. [41] included polyvinyl alcohol fibers in pervious concrete mixtures containing recycled ceramic aggregate. Findings of the study suggest an optimum ceramic substitution rate of 40% can help the compressive and flexural strengths reach 21.35 MPa and 2.74 MPa, respectively, with only a 2.5% reduction in the permeability coefficient from its maximum value. Adding 0.3 volume content of polyvinyl alcohol fibers to the blended recycled pervious concrete mix improved the flexural strength by 24.5%, with negligible effect on the compressive strength and permeability. Results from a study by Mitrosz et al. [42] showed that recycled concrete aggregate with a weight replacement ratio of 50% increased the mechanical properties of PC, while rubber waste aggregate with a volume replacement ratio of 10% reduced the compressive strength by 11.4%. Best results were obtained by adding 2.0 kg/m^3^ of polymer fibers, which was able to increase the strength by up to 25%. A study by Sangthongtong et al. [43] on the mechanical properties of pervious recycled aggregate concrete embedded with sackcloth fibers showed that the compressive strength of the pervious concrete decreased by 40–60% as the void ratio was enlarged from 10 to 30%, irrespective of the size of the aggregates. The use of recycled aggregates did not affect the permeability of pervious concrete made with small-size aggregates with 10% designed air void ratios, keeping it around 7.05 mm/s. Fawzi and Awad [44] investigated the impact of adding polypropylene fiber and silica fume on the mechanical characteristics of pervious concrete containing recycled aggregate. The study showed that the best volumetric percentage of polypropylene fiber was 0.5%, added to 10% replacement of natural aggregate with recycled ones, which increased the tensile and compressive strengths as well as the modulus of elasticity while decreasing the dry density in comparison with conventional concrete mixes. Hailong et al. [45] included fly ash and basalt fibers in pervious concrete mixes containing recycled coarse aggregate made from demolished concrete and brick wastes. Findings of the study suggested an optimal mix combination consisting of 10% fly ash cement replacement and 0.05% basalt volumetric fiber content with 85% recycled concrete aggregate and 15% brick aggregate can greatly improve the mechanical properties, frost resistance and water permeability of pervious concrete.

The previous summary of the literature on the subject suggests a scarcity of research on pervious concrete containing recycled materials and fibers. In particular, no studies exist on the implementation of natural fibers in the form of date palm leaves in pervious recycled concrete mixes. Hence, the study at hand can address this deficiency by filling the gap in the research on this important topic.

## 3. Materials and Methods

**Figure 1:**
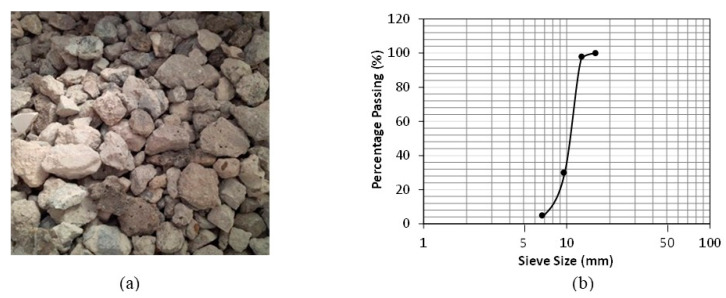
Local RCA used in the study: (**a**) image of aggregate; (**b**) results of sieve analysis.

In this study, the coarse aggregate used to make pervious concrete was obtained from Beeah’s recycling facility in Sharjah, UAE, which is a governmental company that currently accomplishes a diversion rate of more than 76% of the solid municipal, cellulous, construction, marine, metal, plastic, and sewerage waste it receives [46]. The Construction and Waste Demolition Facility part of Beeah in Sharjah, UAE, treats and processes annually around 500,000 metric tons of rubble from construction, renovation, repair, and the demolition of structures and roads. The outcome is in the form of industry-certified recyclable products that can be used as curb stones, interlock tiles, and recycled aggregate. The aggregate is produced in different sizes and shapes, which can be utilized as base or subbase for roads and other structural applications. Upon receiving the concrete rubble from different sites, the waste at the recycling plant is crushed, separated from the metal reinforcement by magnet, manually cleaned from impurities and sieved into different grades based on particle size. From sieve analysis in accordance with ASTM C-136 [47], which is used primarily to determine the grading of materials, the size of the RCA that is used to make pervious concrete in this research varied between 6.73 and 15.9 mm, as shown in Figure 1, with the majority being within the 9–12 mm size range. The particle size distribution for the NA was chosen to closely match that of the RCA.

Visual examination of the recycled coarse aggregate revealed some differences from one batch to another, and when compared with the natural aggregate. While the majority of the recycled aggregate appeared to come from crushed reinforced concrete members, there were some impurities in the form of mortar sticking to the surface of some of the aggregate particles, inferior materials such as fragments of masonry, and debris in the form of tiny wood and plastic pieces.

The specific gravity and water absorption properties for the coarse aggregate employed in the study were determined following the requirements of ASTM C-29 [48] and are presented in Table 1. Note that bulk density values are necessary for selecting proportions for concrete mixtures and finding the mass and volume relations for conversions in purchase agreements. While the obtained specific gravity values for the RCA and NA were close to each other, water absorption for the RCA was higher by 13%. The aggregate was also subjected to Los Angeles abrasion resistance tests according to ASTM C-131 [49] and crushing value tests in conformity with BS 812: Part 110: 1990 [50]. The abrasion test determines the resistance of the aggregate to degradation by scraping and impact, whereas the crushing value test provides a relative measure of the resistance of an aggregate to crushing under a slowly imposed compressive load. For the RCA and NA, the LA abrasion values were, respectively, 31.9% and 24.0%, whereas the crushing values were correspondingly equal to 24.1% and 19.1%. Although both the abrasion and crushing values for the RCA came out inferior to those of the NA, for all practical purposes they are good enough for use in structural concrete since they are either close to the 30% upper limit or below it.

Locally produced Portland cement was used in the mixtures without other cementitious materials such as fly ash or ground granulated blast-furnace slag. The main chemical composition of the cement used in the study consisted of SiO_2_ (20.5%), Al_2_O_3_ (4.7%), Fe_2_O_2_ (4.0%), CaO (64.1%), MgO (1.8%), SO_3_ (2.4%), Na_2_O (0.58%) and LOI (1.5%).

The mix design of the pervious concrete is conducted according to the ACI 522.1, Specification for Construction of Pervious Concrete Pavement [51]. In a typical mix of pervious concrete, the range of the water-to-cement ratio is 0.26–0.4, water is 108–166 kg/m^3^, cement is 270–415 kg/m^3^, coarse aggregate is 1190–1480 kg/m^3^, fine aggregate is 0–1050 kg/m^3^, fine-to-coarse aggregate ratio is 0:1–1:1, and coarse aggregate-to-cement ratio is 4:1–4.5:1. In this study, 12 different mix designs were produced with a water content of 110 kg/m^3^, cement of 356 kg/m^3^, coarse aggregate ranging from 1430 to 1599 kg/m^3^, fine aggregate between 0 and 169 kg/m^3^, and volumetric fiber content in the 0.0–0.7% range. 

**Figure 2:**
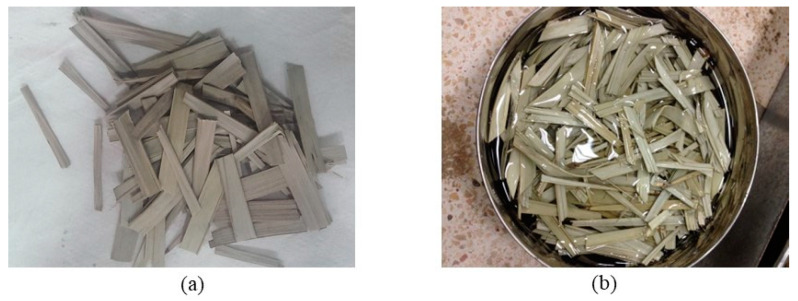
Local date palm leaves fibers used in the study: (**a**) dry; (**b**) soaked.

The natural fibers that were used in the mixes came from the untreated leaves of local date palm trees in the UAE, shown in Figure 2, of which their typical chemical, physical and mechanical properties are taken from the published work of Bamaga [52]. The date palm fibers are typically 60 mm in length and 5–10 mm in width, with density approximately equal to 1.0 g/cm^3^, tensile strength around 39.1 MPa, Young’s modulus roughly about 6.4 GPa, and elongation at breaking point reaching 1.06%. It should be noted that tests on the compressive and tensile strengths of fiber-reinforced pervious concrete in this study, discussed in detail later in the paper, showed that the fibers did not fail in tension even at the onset of collapse of the concrete; instead, they simply de-bonded from the surrounding aggregate before slipping. 

Details of the 12 mix design proportions of the pervious concrete considered in this research are summarized in Table 2. Mix 1 is a control sample that includes coarse natural aggregate, but no coarse recycled aggregate, fine aggregate, or fibers. Mix 2 is another control sample that includes recycled coarse aggregate, but neither fine aggregate nor fibers. Thereafter, each two consecutive pair of mixes (e.g., Mix 3 and Mix 4, or Mix 5 and Mix 6, etc.) mainly differ in the amount of natural fine aggregate and fibers, of which the mix with an odd number does not contain fibers and the one with an even number contains fibers. To illustrate, the ratio of natural sand to recycled coarse aggregate in Mixes 3 (without fibers) and 4 (with 0.09% fibers) is 1.5%. Likewise, the ratio of natural sand to recycled coarse aggregate in Mixes 5 (0% fibers) and 6 (with 0.18% fibers) is 3.1%, in Mixes 7 (0% fibers) and 8 (0.28% fibers) is 4.7%, in Mixes 9 (0% fibers) and 10 (0.46% fibers) is 8.1%, and in Mixes 11 (0% fibers) and 12 (0.64% fibers) is 11.7%. The ratio of the weight of fine aggregate to that of natural fibers in the mixes that contain both ingredients was almost constant, varying between 26.09 and 26.97. The mass density of the fresh concrete varied within a narrow range of 2065–2071 kg/m^3^. The mix designation has the form RX-FA/CAY-Z, in which X denotes the recycled aggregate replacement percentage with regard to the natural coarse aggregate (X = 0 or 100), Y denotes the natural fine aggregate to coarse aggregate percentage (Y = 0–11.7%), and Z denotes the volumetric percentage of the date leaves fibers in the concrete mix (Z = 0–0.64%).

**Figure 3:**
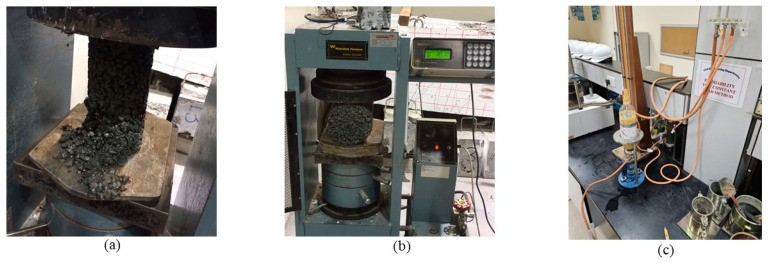
Tests conducted on the hardened pervious concrete: (**a**) axial compression; (**b**) split tensile; (**c**) permeability.

To improve the bond between the aggregate and cement paste, a small amount of cement (<5% by mass) with coarse aggregate was dry mixed for 1 min until completely coated before adding the water. Afterwards, the remaining cement, water and other materials were added and mixed for 3 min, allowed to rest for another 3 min and then mixed for an additional 2 min before casting in the cylindrical molds. The compaction on the molded specimens was conducted using a vibrating table where the mixture was placed in three layers and each layer was compacted by vibration for 5 s. The use of tamping and rodding for compaction was avoided because it was difficult to utilize in such low slump pervious concrete, it could have damaged the date palm fibers within the mix, and such an action could have disturbed the uniform dispersion of the fibers within the concrete mixture. After 24 h, the samples were de-molded and cured for a week according to ASTM C-192 [53]. Each of the considered concrete mixtures was cast into six 150 × 300 mm cylinders and three 100 × 200 mm cylinders, and the reported results in this paper represent the average values of 3 samples for each measured variable. The large cylinders were used for the split cylinder tensile according to ASTM C39 [54] and axial compressive strength according to ASTM C496 [55] tests, while the small cylinders were utilized in the permeability tests. The strength and permeability tests were conducted on the hardened concrete samples after 28 and 20 days from the day of casting, respectively. Figure 3 shows pictures from the tests on the concrete.

The compressive strength of the pervious concrete was determined using a compression machine by determining the maximum stress that the cylindrical specimens can support while standing up on its base prior to collapse at a loading rate of 0.25 MPa/second. The split cylinder tensile strength of the concrete was found by applying a load on the cylinder while it is sitting on its side through bearing strips at a rate equal to 0.1 MPa/second.

To prepare concrete samples for the permeability tests, the 100 × 200 mm cylinders were incased in nylon with the insides submerged in silicone and then wrapped in tape to secure them and to prevent water from leaking from the sides of the cylinders. The top part of plastic drinking water bottles was then wrapped around the top and bottom of the cylinders to allow water to go through the intake to the cylinder and out without dripping. The permeability of the specimens was determined using the falling head test scheme. The average coefficient of permeability (k) for each of the 12 concrete mixes considered was then determined for 700 mm water fall following Darcy’s law and assuming laminar flow. 

## 4. Results

In this section, results from the experimental testing of the hardened concrete considered in the study regarding the compressive strength, split tensile strength, permeability and porosity are provided. The effects of using recycled coarse aggregate, adding natural fine aggregate and incorporating date palm leaves fibers in the concrete mixtures on the properties of pervious concrete are included and discussed. 

### 4.1. Compressive Strength

The compressive strength of 150 mm × 300 mm concrete cylinders at the age of 28 days is determined by concentrically loading the specimens along a line perpendicular to its circular cross-section while being placed vertically on its base inside a compression machine. The test results are shown in Table 3.

The effect of replacing the natural coarse aggregate with recycled aggregate on the concrete compressive strength is obtained by comparing mixes 1 and 2 in Table 3. The tests showed that 100% replacement of the aggregate caused the compressive strength of pervious concrete to reduce by 36%, from 7.08 to 4.53 MPa. Earlier studies by the first two authors [56,57,58,59,60,61] on non-pervious concrete have indicated the full replacement of natural coarse aggregate with recycled aggregate from construction demolition waste obtained from the same source can reduce the compressive strength by an average of just 10–15%. The lack of adequate amount of fine aggregate in pervious concrete increases the importance of the coarse aggregates and the bond between them to the mechanical properties of the concrete. Hence, the quality of the coarse aggregate, including its strength, surface texture and bond characteristics, can have drastic consequences on the compressive strength of pervious concrete. The lower abrasion results (24% versus 31.9%) and crushing values (19.1% versus 24.1%) for the recycled aggregate compared to natural aggregate have contributed to the decrease in compressive strength of the pervious concrete.

**Figure 4:**
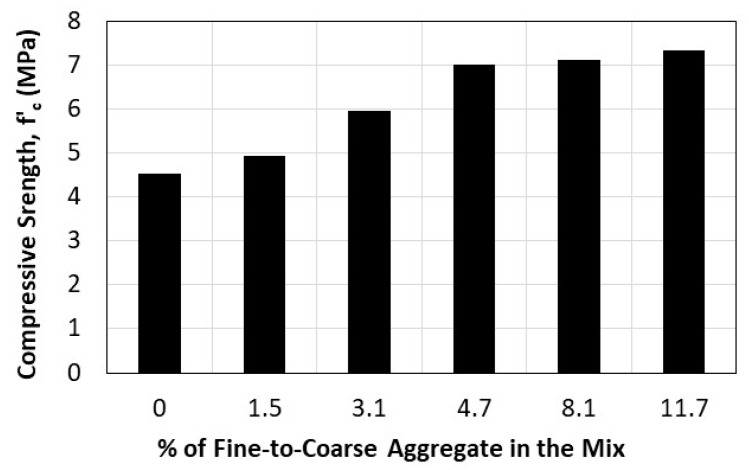
Effect of adding fine aggregate on the compressive strength of pervious concrete.

To understand the effect of adding fine aggregate to pervious concrete mixtures on concrete strength, we examine the results of Mixes 2, 3, 5, 7, 9, and 11, which contain fine aggregate in varying amount (FA/RCA = 0–11.7%), without fibers. The findings are presented in Figure 4, which shows the effect of incorporating fine aggregate into pervious concrete mixes. Note that the impact of adding fine aggregate on compressive strength is most significant when the fine-to-coarse aggregate percentage is less than 5%. Beyond the 5% limit, any added quantity of fine aggregate has negligible consequence on strength. For reference, Mix 1, which contains natural coarse aggregate without sand, has a compressive strength of 7.08 MPa, a level of strength which the concrete mixes with at least 4.7% fine-to-coarse aggregate were able to attain while containing the recycled aggregate. 

**Figure 5:**
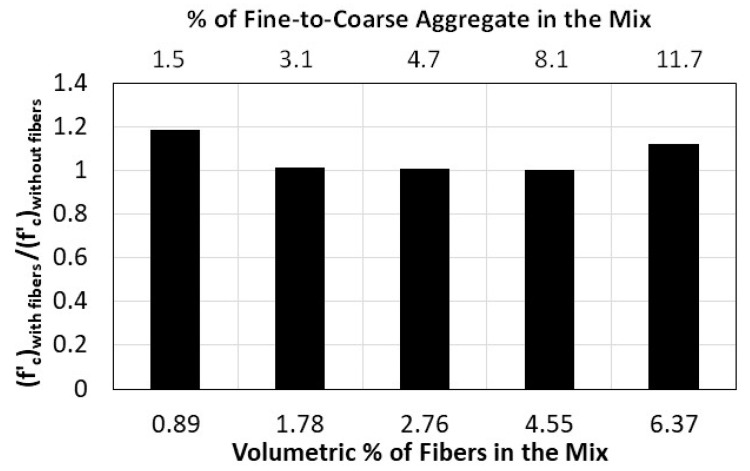
Effect of adding palm date leaves fibers on the compressive strength of pervious concrete.

Experience has shown that fiber addition to concrete mixes can have a positive effect on the concrete by averting sudden failure, improving the fracture energy, decreasing crack width, lessening volumetric changes with time, and increasing tensile strength. The effect of adding natural date palm leaves fibers on the compressive strength of recycled pervious concrete is demonstrated in this study by comparing the outcomes of mixes 3 and 4, mixes 5 and 6, and so on. The odd-numbered mixes contain no fibers while the even-numbered mixes include fibers. The volumetric ratio of the fibers in the concrete mixtures that contain fibers varies between 0.09% and 0.64%. While such percentages seem low, adding more fibers can adversely affect the workability of the fresh mix and bonding between the aggregate. Note that in this study, the addition of fibers is simultaneously accompanied with adding larger amounts of fine aggregate since higher volumetric ratios of fibers require more paste for the fibers to be anchored within; otherwise, they will slip from the aggregate while under high internal forces. Although the results of the study, shown in Figure 5, indicate a 0.4–18.6% improvement in compressive strength with the addition of fibers, there is no clear relationship between the percentage of volumetric fiber content and compressive strength. While the compressive strength improves by 18.6% with the addition of 0.89% of fibers and by 12% with the addition of 6.37% of fibers, the enhancement in compressive strength was negligibly small with the addition of 1.78%, 2.76% and 4.55% of fibers. This finding suggests more tests are needed before reaching a reliable conclusion and the finding could be the result of changing two variables, quantity of fine aggregate and amount of fibers, at the same time.

### 4.2. Split Tensile Strength

A split tensile strength test on a concrete cylinder is an indirect method to determine the tensile strength of concrete since the direct tension test has multiple drawbacks, such as unintended load eccentricity, strain nonuniformity and stress concentration at the specimen ends. The split tensile strength of 150 mm × 300 mm concrete cylinders at the age of 28 days is determined by loading the specimens along its length while being placed on its side inside a compression machine. The test results are shown in Table 4.

The influence of substituting the natural coarse aggregate with recycled aggregate on the split tensile strength of the pervious concrete is determined by comparing mixes 1 and 2 in Table 4. The experimental test results demonstrate that the tensile strength of pervious concrete drops from 1.56 MPa when natural aggregate is used to 0.67 MPa when recycled aggregate is used. Although concrete employing recycled aggregate is expected to be weaker in tension when compared with concrete containing natural aggregate, the observed drop is much more drastic when compared with previous studies by the authors [56,57,58,59,60,61] on non-pervious concrete. The inferior quality of the recycled coarse aggregate due to its higher water absorption, lower density, possible content of organic substances, higher level of crushability, and reduced abrasion resistance contributes to lower tensile strength of pervious concrete. 

**Figure 6:**
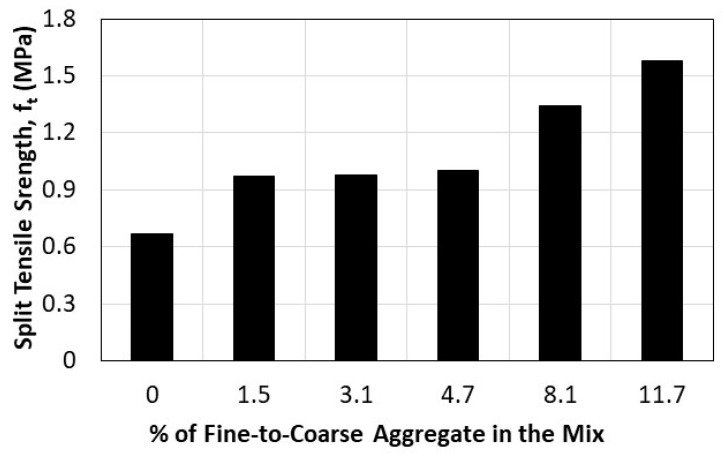
Effect of adding fine aggregate on the split tensile strength of pervious concrete.

To quantify the impact of adding fine aggregate to pervious concrete mixtures on their tensile strength, we compare the results of Mixes 2, 3, 5, 7, 9, and 11, each of which include fine aggregate in varying amounts (FA/RCA = 0–11.7%), without fibers. The results that are shown in Figure 6 indicate the positive effect of incorporating fine aggregate into pervious concrete mixes on tensile strength. Even a small amount of fine aggregate equal to 1.5% of the coarse aggregate can enhance the tensile strength by 45% compared to that of concrete containing just recycled coarse aggregate. Adding a percentage of fine aggregate equivalent to 11.7% of the coarse aggregate into a pervious concrete mix can help restore the tensile strength to that of pervious concrete made with 100% natural aggregate.

**Figure 7:**
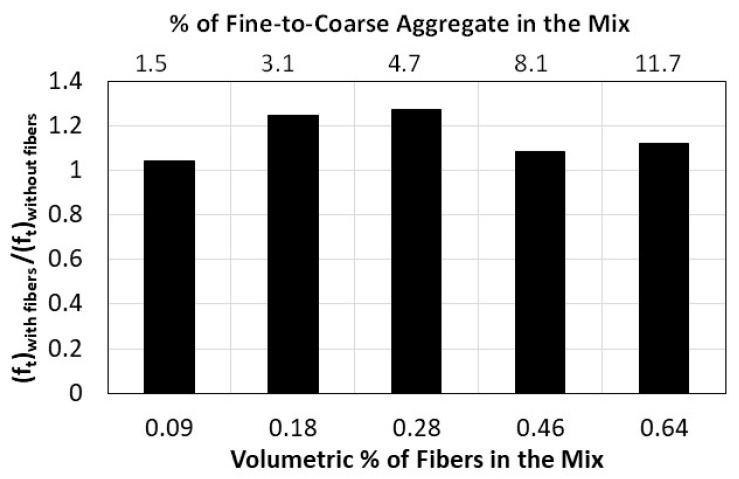
Effect of adding palm date leaves fibers on the split tensile strength of pervious concrete.

Published research on the incorporation of fibers into concrete mixes has consistently demonstrated the effectiveness of the fibers in bridging cracks caused by internal tensile stresses within the concrete, which not only increases the tensile strength of the material but also its ductility. The effect of adding natural date palm leaves fibers on the split tensile strength of recycled pervious concrete is shown in this study by comparing the strength of odd-numbered mixes (≥3) that do not contain fibers with their corresponding even-numbered mixes (≥4) that contain fibers. Figure 7 shows that adding natural fibers to pervious concrete mixes in volumetric ratios of 0.09–0.64% can improve the tensile strength by 4–24%, with an average increase of 15%. For significant improvement in tensile strength, the volumetric percentage ratio should be at least 0.18%. 

**Figure 8:**
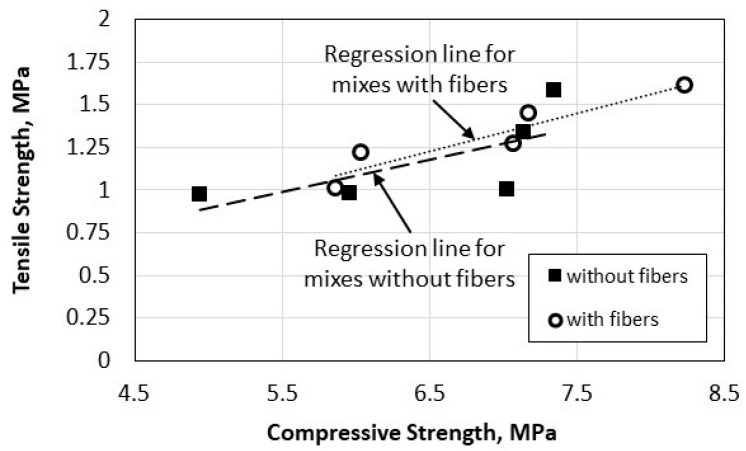
Relationship between tensile and compressive strengths of recycled aggregate pervious concrete.

The relationship between the tensile and compressive strengths for the 10 pervious recycled concrete mixes that contain natural sand and fibers (Mixes 3–12) is presented in Figure 8. As expected, there is an increase in the tensile strength with an increase in the compressive strength, especially for the mixes that contain date palm leaves fibers. The effect of the natural fiber and sand content on the mathematical relationship between the tensile and compressive strengths is included in Section 5.

### 4.3. Density, Permeability and Porosity

In this section, the density, *ρ*, permeability, K, and porosity, Φ, of pervious concrete containing sand and fibers is investigated. Note that permeability is the rate of flow of fluid through concrete under a pressure gradient, whereas porosity is a measure of the volume of voids within the concrete. Both properties are affected by the density of the material. Table 5 presents the results of those parameters for the five mixtures that contain fibers (and fine natural aggregate), denoted by Mixes 4, 6, 8, 10 and 12. For comparison, the parameters of Mix 2, which contains no fibers, are also included in the table. As explained earlier, in this study, the amount of natural fine aggregate is increased with the rise in the percentage of fibers in the mixes.

**Figure 9:**
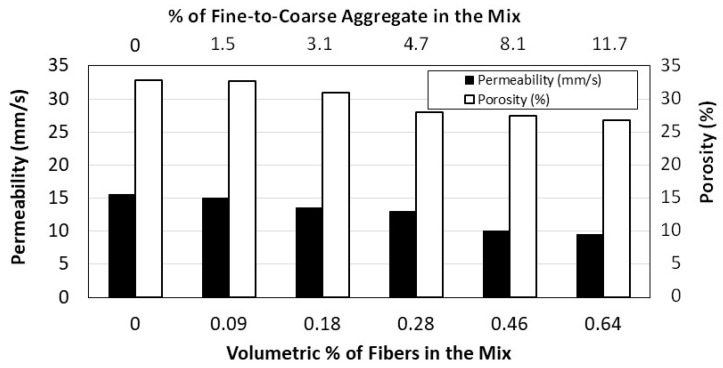
Effect of fine aggregate and fibers on the permeability and porosity of pervious concrete.

As anticipated, the density of the concrete increases with the addition of the fine aggregate, from 1763 kg/m^3^ for Mix 2 without fine aggregate to 1947 kg/m^3^ for Mix 12 that contains fine aggregate equivalent to 11.7% of the coarse aggregate. Compared to traditional concrete, the considered pervious concrete in this study can be labeled as lightweight, since its mass density is way below that of normal weight concrete (about 2300 kg/m^3^). The increase in the amount of fine aggregate also impedes the flow of water through the concrete due to the decrease in the amount and size of voids within the concrete. Although the presence of date palm leaves fibers in the mixtures has minimal impact on the density, it has some influence on the permeability and porosity, which in this study is difficult to assess since the quantity of fibers and amount fine aggregate were combined in the mixes. Figure 9 shows the decline in permeability and porosity with the increase in fine aggregate in the mixes that contain fibers.

## 5. Analysis of Results

In this section, the results presented earlier on the compressive strength, split tensile strength, density, permeability and porosity for the even-numbered mixes are further analyzed and potential correlations between the parameters are explored. Note that except for Mix 2, which does not contain fibers or fine aggregate, all other mixes contain fine aggregate and fibers of various amount, such that the ratio of the weight of the fine aggregate to that of fibers is kept almost constant (between 26.09 and 26.97).

With regard to the strength parameters, the ACI 318 code [61] includes an equation that predicts the split tensile strength of conventional Portland cement concrete, *f_t_* (MPa), from the compressive strength, $f_{c}^{\prime }$ (MPa), as follows:

(1) $$f_{t}=0.56\lambda \sqrt{f_{c}^{\prime }}$$

in which *λ* is the lightweight concrete factor, which for concrete that has mass density, *w_c_*, between 1600 and 2160 kg/m^3^ can be obtained from:

(2) $$\lambda =0.00047w_{c}\leq 1.0$$

By modifying Equation (1) to include factors that account for the fine-to-coarse aggregate fraction, FA/RCA, volumetric fiber content in the mix, *V_f_*, and suitable constants, the following expression of the tensile strength as function of the compressive strength, $f_{c}^{\prime }$ (MPa), is obtained:

(3) $$f_{t}=0.4\;\lambda \left(1+5V_{f}\right)\left[1+½\left(FA/RCA\right)\right]\sqrt{f_{c}^{\prime }}$$

**Figure 10:**
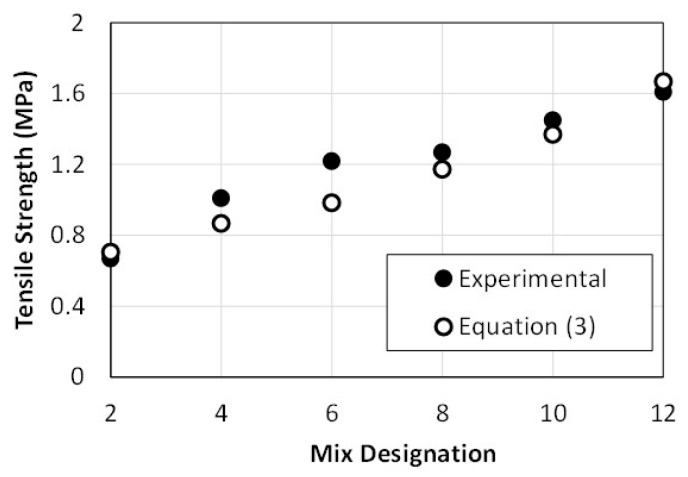
Validation of the proposed split tensile strength predictive equation.

Validation of the proposed equation shown above for pervious concrete made with recycled concrete aggregate, fine aggregate and palm leaves fibers is illustrated in Figure 10. In the figure, (*f_t_*)*_Theo_* is the tensile strength obtained by Equation (3) and (*f_t_*)*_Exp_* is the tensile strength obtained from the experimental testing. The outcome indicates that Equation (3) can reasonably predict the tensile strength of the pervious concrete considered in the study from the compressive strength, with a practical degree of conservatism.

**Figure 11:**
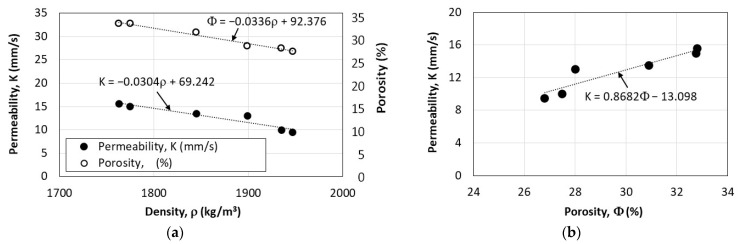
Relationships between parameters of pervious concrete: (**a**) effect of density on permeability and porosity; (**b**) relationship between porosity and permeability.

The correlation of permeability and porosity with the density of fiber-reinforced pervious recycled aggregate concrete is presented in Figure 10. The rate of decrease of permeability and porosity with the increase in density is nearly equal, as shown in Figure 11a. This is an expected outcome since permeability and porosity are highly related, as shown in Figure 11b. 

**Figure 12:**
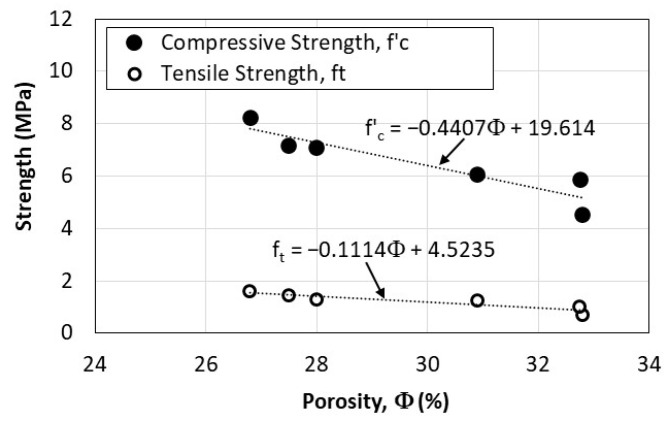
Effect of porosity on compressive and split tensile strengths.

The influence of porosity on strength properties of the fiber-reinforced pervious concrete considered in the study is shown in Figure 12. While it is expected that both tensile and compressive strengths of concrete decrease with the increase in porosity, the results indicate that the compressive strength is more impacted by the porosity than the tensile strength. This is attributed to fact that the presence of fibers in concrete mixtures affects the tensile strength more than the compressive strength.

## 6. Conclusions

Results of this study on recycled pervious concrete that are reinforced with date palm leaves fibers lead to the following conclusions:

1. 100% replacement of natural coarse aggregate with recycled coarse aggregate from concrete demolition waste in pervious concrete mixes can reduce the compressive strength by 36% and the tensile strength by 57%.
2. The addition of natural fine aggregate can help increase the density and enhance compressive and tensile strengths of pervious concrete. Adding fine aggregate equivalent to 11.7% of the recycled coarse aggregate in pervious concrete mixes can help restore the compressive and tensile strengths of the pervious concrete to the same level of pervious concrete made with 100% natural aggregate and without fine aggregate.
3. While the influence of adding date palm leaves fibers to pervious concrete made with recycled coarse aggregate is minimal on the compressive strength, its effect on the tensile strength is reasonably high.
4. The addition of fine natural aggregate and date palm leaves fibers in uniform amounts to pervious concrete mixtures that incorporate recycled coarse aggregate reduces the permeability and porosity of the hardened product.
5. The tensile strength of pervious concrete utilizing recycled aggregate and natural aggregate and reinforced with date palm leaves fibers can be predicted from the compressive strength.
6. There is a strong relationship between permeability and porosity since both parameters are approximately linearly correlated with the density of the concrete material.
7. The impact of the porosity of pervious concrete made with recycled aggregate and containing date palm leaves fibers is more pronounced on the compressive strength than it is on the tensile strength.

Overall, results of the study demonstrated the feasibility of using locally produced recycled concrete coarse aggregate from demolition waste and the positive potential of implementing natural fibers from date palm leaves in pervious concrete mixes. While this study concentrated on the short-term mechanical properties and permeability aspects of the sustainable material, future studies on the subject may consider the long-term characteristics and durability attributes.

## Figures and Tables

**Table 1:** Specific gravity and absorption properties of the RCA and NA used in the study.

Type of Aggregate	Bulk Specific Gravity (Dry)	Saturated-Surface-Dry Bulk Specific Gravity	Apparent Specific Gravity	Water Absorption
RCA	2.464	2.576	2.774	4.53%
NA	2.440	2.540	2.700	3.99%

**Table 2:** Pervious concrete mixture proportions for the 12 considered mixes.

Weight of Ingredients (kg/m^3^)	Mix Number and Designation											
	1	2	3	4	5	6	7	8	9	10	11	12
	R0-FA/CA0-F0	R100-FA/CA0-F0	R100-FA/CA1.5-F0	R100-FA/CA1.5-F0.09	R100-FA/CA3.1-F0	R100-FA/CA3.1-F0.18	R100-FA/CA4.7-F0	R100-FA/CA4.7-F0.28	R100-FA/CA8.1-F0	R100-FA/CA8.1-F0.46	R100-FA/CA11.7-F0	R100-FA/CA11.7-F0.64
Water	110	110	110	110	110	110	110	110	110	110	110	110
Portland Cement	356	356	356	356	356	356	356	356	356	356	356	356
Natural Coarse Aggregate	1599	0	0	0	0	0	0	0	0	0	0	0
Recycled Coarse Aggregate	0	1599	1575	1575	1551	1551	1527	1527	1479	1479	1431	1431
Natural Fine Aggregate % FA/CA	0 0	0 0	24 1.5%	24 1.5%	48 3.1%	48 3.1%	72 4.7%	72 4.7%	120 8.1%	120 8.1%	168 11.7%	168 11.7%
Natural Fibers % by Vol.	0 0%	0 0%	0 0%	0.89 0.09%	0 0%	1.78 0.18%	0 0%	2.76 0.28%	0 0%	4.55 0.46%	0 0%	6.37 0.64%

**Table 3:** Compressive strength results for the pervious concrete mixes considered in the study.

Parameter (MPa)	Mix Number and Designation											
	1	2	3	4	5	6	7	8	9	10	11	12
	R0-FA/CA0-F0	R100-FA/CA0-F0	R100-FA/CA1.5-F0	R100-FA/CA1.5-F0.09	R100-FA/CA3.1-F0	R100-FA/CA3.1-F0.18	R100-FA/CA4.7-F0	R100-FA/CA4.7-F0.28	R100-FA/CA8.1-F0	R100-FA/CA8.1-F0.46	R100-FA/CA11.7-F0	R100-FA/CA11.7-F0.64
Compressive Strength	7.08	4.53	4.94	5.86	5.96	6.04	7.03	7.07	7.14	7.17	7.34	8.23

**Table 4:** Split tensile strength results for the pervious concrete mixes considered in the study.

Parameter (MPa)	Mix Number and Designation											
	1	2	3	4	5	6	7	8	9	10	11	12
	R0-FA/CA0-F0	R100-FA/CA0-F0	R100-FA/CA1.5-F0	R100-FA/CA1.5-F0.09	R100-FA/CA3.1-F0	R100-FA/CA3.1-F0.18	R100-FA/CA4.7-F0	R100-FA/CA4.7-F0.28	R100-FA/CA8.1-F0	R100-FA/CA8.1-F0.46	R100-FA/CA11.7-F0	R100-FA/CA11.7-F0.64
Split Tensile Strength	1.56	0.67	0.97	1.01	0.98	1.22	1.00	1.27	1.34	1.45	1.58	1.61

**Table 5:** Density, permeability and porosity of some of the mixes considered in the study.

Parameter		Mix Number and Designation				
	2	4	6	8	10	12
	R100-FA/CA0-F0	R100-FA/CA1.5-F0.09	R100-FA/CA3.1-F0.18	R100-FA/CA4.7-F0.28	R100-FA/CA8.1-F0.46	R100-FA/CA11.7-F0.64
Density (Kg/m^3^)	1763	1775	1845	1899	1935	1947
Permeability (mm/s)	15.6	15.0	13.5	13.0	10.0	9.5
Porosity (%)	32.81	32.75	30.90	28.00	27.50	26.80

## Data Availability

All data are contained within the article.
